# 4Ps medicine of the fatty liver: the research model of predictive, preventive, personalized and participatory medicine—recommendations for facing obesity, fatty liver and fibrosis epidemics

**DOI:** 10.1186/1878-5085-5-21

**Published:** 2014-12-07

**Authors:** Francesca Maria Trovato, Daniela Catalano, Giuseppe Musumeci, Guglielmo M Trovato

**Affiliations:** Department of Clinical and Experimental Medicine, Internal Medicine Division, School of Medicine, University of Catania, Via S. Sofia, 78-95123 Catania, Italy; Department of Biomedical and Biotechnological Sciences, Human Anatomy and Histology Section, School of Medicine, University of Catania, Via S. Sofia, 87-95123 Catania, Italy

**Keywords:** Adipose tissue, Diet, Fatty liver, Liver fibrosis, Obesity, Lifestyle, Fashion, Suboptimal Health, Interactome

## Abstract

Relationship between adipose tissue and fatty liver, and its possible evolution in fibrosis, is supported by clinical and research experience. Given the multifactorial pathogenesis of non-alcoholic fatty liver disease (NAFLD), treatments for various contributory risk factors have been proposed; however, there is no single validated therapy or drug association recommended for all cases which can stand alone. Mechanisms, diagnostics, prevention and treatment of obesity, fatty liver and insulin resistance are displayed along with recommendations and position points. Evidences and practice can get sustainable and cost-benefit valuable outcomes by participatory interventions. These recommendations can be enhanced by comprehensive research projects, addressed to societal issues and innovation, market appeal and industry development, cultural acceptance and sustainability. The basis of participatory medicine is a greater widespread awareness of a condition which is both a disease and an easy documented and inclusive clue for associated diseases and unhealthy lifestyle. This model is suitable for addressing prevention and useful for monitoring improvement, worsening and adherence with non-invasive imaging tools which allow targeted approaches. The latter include health psychology and nutritional and physical exercise prescription expertise disseminated by continuous medical education but, more important, by concrete curricula for training undergraduate and postgraduate students. It is possible and recommended to do it by early formal teaching of ultrasound imaging procedures and of practical lifestyle intervention strategies, including approaches aimed to healthier fashion suggestions. Guidelines and requirements of research project funding calls should be addressed also to NAFLD and allied conditions and should encompass the goal of training by research and the inclusion of participatory medicine topics. A deeper awareness of ethics of competences in health professionals and the articulation of knowledge, expertise and skills of medical doctors, dieticians, health psychologists and sport and physical exercise graduates are the necessary strategy for detectin a suboptimal health status and achieving realistically beneficial lifestyle changes. “The devil has put a penalty on all things we enjoy in life. Either we suffer in health or we suffer in soul or we get fat” (Albert Einstein); the task of medical research and intervention is to make possible to enjoy life also without things that make sufferance in health and souls and which excessively increase body fat.

## Review

### Introduction

#### Overview on adipose tissue

##### Ancient bias

A good woman had a hen that laid her every day an egg. Then, the woman had an idea. “I will feed the hen twice as much food so that she will lay twice as many eggs!” Instead of one bowl of food for breakfast, the woman gave the hen two bowls of food for breakfast every day. Soon, the hen became so fat that it became too lazy to make any more eggs. So, the woman had no egg anymore (Aesop, 620–564 BCE). Also, the appreciated foie gras, the liver of a duck or goose that has been specially fattened, has a similar history becoming an unhealthy food from an unhealthy animal, sacrificed in honour of gastronomy.

Despite the obvious fundamental truth, from the fat to the liver, to fatty liver and behind, there is a jump, with likely prejudices of oversimplification. Epidemiological and interventional evidences outline relationship and likely mechanisms between peripheral fat excess and vital organ damage: this is particularly relevant for liver, blood vessels and heart. Relationship and association are not proof of causality, and fat content inside adipocytes is quite different from fat content in liver and in other cells. The prompt availability of cheap and high caloric food is a relatively recent phenomenon, and although it involves especially the Western countries, it is increasing in a worldwide fashion.

The smallest molecules with the greatest energy content are fatty acid, and the cell specialized in their storage are adipocytes of the white fat. This tissue has the capacity to accumulate and release rapidly fatty acids and to store them as triglycerides (TG): in particular situation, such as obesity, adipocytes hold in their cytoplasm TG and increase their volume by six to seven times [1]. Furthermore, adipose tissue communicates with other organs by secreting hormones, commonly referred to as adipokines that mediate the molecular cross-talk that occurs among adipose tissue, muscle and liver. Among these, leptin increases free fatty acid delivery from adipose tissue to the liver. Differently, circulating adiponectin is inversely correlated with body fat percentage, promotes insulin sensitivity and increase glucose utilization and free fatty acid oxidation in the liver [2]. Hypoadiponectinemia together with other cytokines, i.e. tumour necrosis factor-alpha (TNF-α) or plasminogen activator inhibitor-1 (PAI-1), induced by the accumulation of visceral adipose tissue (VAT) might be a major background of metabolic disorders, including insulin resistance (IR) and metabolic syndrome (MS) [3]. Although the ability to contain TG is uncommon in mammalian cells, adipocytes share it with hepatocytes and striated muscle cells. It is well established that variable amounts of TG are found in the liver of healthy subjects [1].

#### Hepatic steatosis or fatty liver

Hepatic steatosis or fatty liver is a disease in which lipids accumulate in the hepatocyte’s cytoplasm with, usually, an increase in volume of the liver. If alcohol consumption is excluded (less than 20 g/die for women and less than 40 g/die for men), the term we use is non-alcoholic fatty liver disease (NAFLD). The prevalence of NAFLD is around 20%–35% in the general population, and an increasing number of patients presents risk factor for its development [4, 5]; in obese people, instead, prevalence is about 75% [6]. Asian populations are also experiencing increased prevalence of Western diseases due to westernized pattern in diet and to a shift toward more unhealthy lifestyle. It is not a prerogative of adults, since epidemiological data show that NAFLD affects up to 10% of children, more frequently boys, and prevalence increases with age. NAFLD is rare in children aged less than 10 years and usually presents with overweight or obesity [7]. Paediatric NAFLD should not be underestimated; in fact, a 20-year retrospective longitudinal cohort study reported that some children progressed to advanced fibrosis or cirrhosis during follow-up, and others developed end-stage liver disease with the consequent liver transplantation. Furthermore, NAFLD is associated with a significantly shorter long-term survival as compared to the expected survival of the general population [8]. Western diet and overnutrition is a common cause of this disease, but not the only one. NAFLD is a multifactorial disorder that rises from a combination of risk factors, such as hypertriglyceridemia, obesity and insulin-resistance (primary NAFLD), but there are also other causes (secondary NAFLD) such as infectious diseases [9, 10]. Hepatitis C virus (HCV), mainly genotype 3, interferes with fat deposition in the liver impairing secretion and degradation and increasing the synthesis of lipids; furthermore, fatty liver is significantly reduced or disappears after a successful antiviral therapy [11]. Nonetheless, adenovirus infection, in particular Ad-36, increases fat storage in adipose tissue but seemingly exerts a protective effect against NAFLD [12, 13]. Ad-36 shows adipogenic but also anti-diabetic properties; it up-regulates peroxisome proliferator-activated receptor gamma (PPARγ) while increasing glucose uptake. *In vitro* studies demonstrated that in particular, the E4orf1 protein of Ad-36 plays a role in the enhanced glucose disposal and was proposed as a therapeutic agent for NAFLD since it could exert its effect on hepatocyte metabolism [14]. Furthermore, another virus, i.e. Ad-37, that increases adiposity in animals and is associated with obesity in humans too is significantly associated with NAFLD in non-diabetic patients on an appropriate diet, and it was hypothesized as adjunctive hallmark of an unfavourable clinical-metabolic profile, if not a causative factor of NAFLD [15].

**Figure 1:**
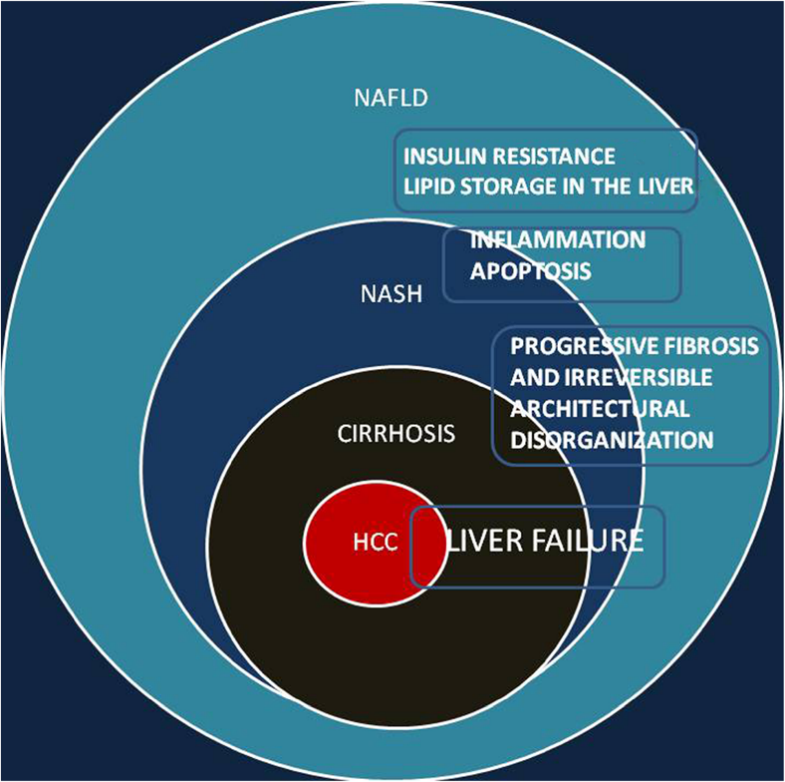
**Relative prevalence of disease from fatty liver to hepatocarcinoma; the most likely associated mechanisms.**

**Figure 2:**
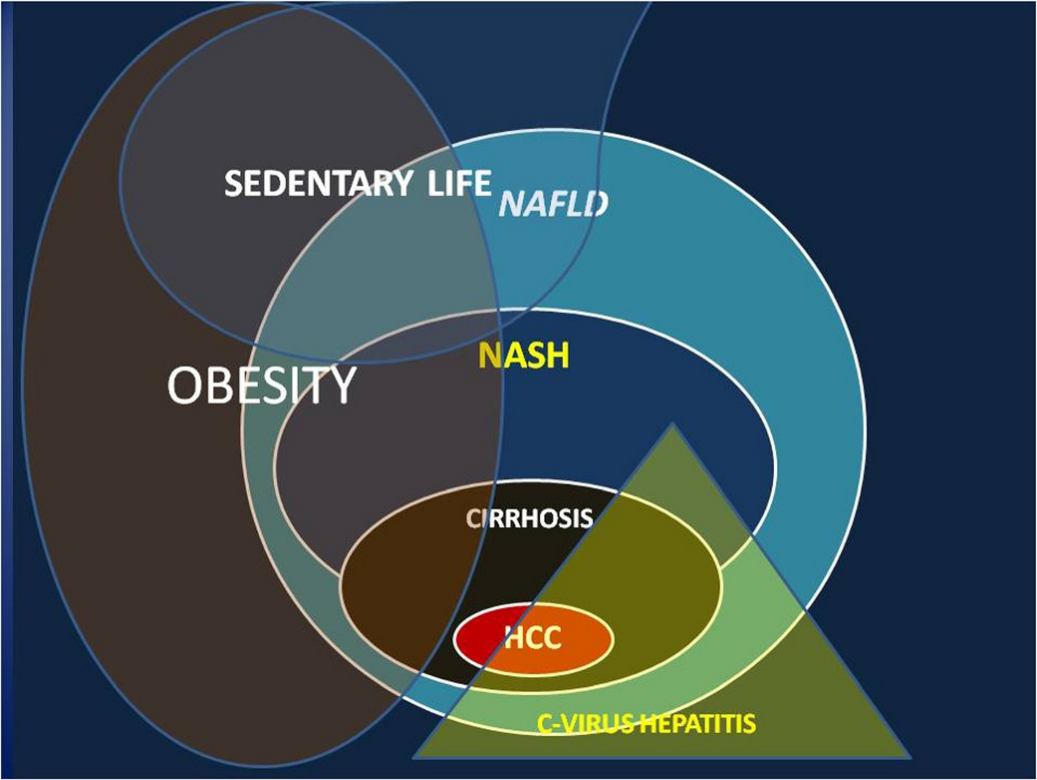
**Cause and co-factors leading fatty liver to HCC.**

Other causes, although less frequent, are noteworthy, such as rare disorder of lipid metabolism inherited in a Mendelian fashion (abetalipoproteinemia, hypobetalipoproteinemia or lipodystrophy), glycogen storage disease or nutritional state such as starvation, total parenteral nutrition or severe surgical weight loss. The hypothesis that genetic profile is involved in the pathogenesis of NAFLD is strengthened by the variability of incidence in different ethnic populations, suggesting that heritability and/or environmental factors influence the disease development. Polymorphisms in various genes affecting lipid metabolism, oxidative stress, IR and immune regulation have been studied as risk factors to the development of hepatic steatosis, but no one is sufficient to explain the complex pathogenesis of this disease [16]. It is known that, despite these evidences, the use of genetics as a tool for predicting NAFLD is still far from being established. NAFLD has a heritable component to susceptibility, a complex trait resulting from environmental exposures acting on a susceptible polygenic background and comprising multiple independent modifiers. Approximately 10 million single nucleotide polymorphisms (SNPs) for which both possible alleles have a population frequency of >1% are present in the human genome. Different genome-wide association studies (GWAS) on NAFLD were published since 2008, identifying a significant association with increased hepatic TG levels for the SNPs of the *PNPLA3* gene [17]. Patatin-like phospholipase domain-containing 3 (PNPLA3) is an enzyme encoded by the *PNPLA3* gene related to adipose triglyceride lipase, the major TG hydrolase in adipose tissue. Although other authors showed that higher NAFLD activity scores were associated with a variant in farnesyl diphosphate farnesyl transferase 1, an enzyme with a role in cholesterol biosynthesis [18], PNPLA3 represents the only gene that has been consistently identified across multiple GWAS as a potential modifier of both hepatic TG accumulation and clinical biochemistry indices as raised serum alanine aminotransferase (ALT) levels. In addition, one variant *PNPLA3* gene, S453I, was found to be common in African Americans compared with European Americans and Hispanics and it was associated with a lower hepatic fat content confirming the epidemiological evidence [19]. In some cases, medications may cause liver steatosis, for example, highly active antiretroviral therapy, amiodarone, methotrexate or tamoxifen. Finally, patients with celiac disease often show elevated transaminase levels and they have an increased risk to develop fatty liver [20]. In the attempt to find single biomarkers useful as predictive tools for liver disease and NAFLD, research studies were addressed to its genetic profile. When steatosis is associated with detrimental inflammatory cascade (approximately 10%–25% of patients with NAFLD), due to severe hepatic lipotoxicity, this condition in named non-alcoholic steatohepatitis (NASH) and it can progress to cirrhosis and, in some cases, to hepatocarcinoma (Figure 1); the interaction of other factors, including lifestyle features, is equally relevant (Figure 2). Free fatty acids (FFA), in particular saturated and not their esterified product (TG) are responsible for liver damage through hepatocyte apoptosis (lipoapoptosis), and FFA serum levels correlate with the severity of the disease [21]. Liver is responsible of the energy homeostasis, playing a central role in the storage of glucose as glycogen and in the distribution of glucose and fatty acid as energy fuels for the other organs. The intrahepatic accumulation of lipid droplets can be summarized in these simple steps: 1) increased uptake of lipids, 2) elevated *de novo* synthesis of fatty acids, 3) impaired lipoprotein synthesis or secretion and/or 4) reduced fatty acid oxidation [22]. Hepatic glucose and FFA uptake occurs in an insulin-dependent way; after meals, lipids are transported as chylomicrons from the gut to the liver through the lymphatic system. Here, they are stored, processed and assembled with apolipoprotein B100 (ApoB) to form very-low-density lipoprotein (VLDL) and then secreted to be stored as lipids in the adipose tissue [23]. The liver does not typically function to store excessive amounts of lipids as energy reserves for the body. When a dietary overload occurs, as in the high-fat and high-carbohydrates western diet, it may result in hepatic lipid accumulation, due also to the *de novo* fatty acid synthesis from carbohydrate metabolism through acetyl-CoA. In NAFLD patients, there is an elevated level of circulating FFA, probably due to loss of sensitivity to insulin in the adipose tissue and to defect in the suppression of lipolysis [24]. Then, there is a shift from lipolysis to *de novo* fatty acid synthesis in livers of insulin-resistant people.

**Figure 3:**
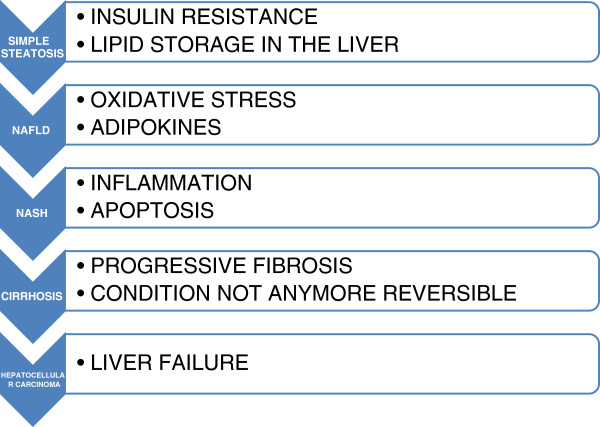
**Fatty liver pathogenesis.** Representation of the step-by-step progression of the fatty liver pathogenesis, with its respective pathologic features.

Initially, there is a reversible intracellular deposition of lipids that leads, through molecular modifications, to oxidative stress and cytokine-induced liver damage, due also to Kupffer cell activation responsible for oxidative stress defense and pro-inflammatory cytokine release. Elevated reactive oxygen species (ROS) lead to organelle toxicity, suppression of fatty acid oxidation and inhibition of lipoprotein assembly and secretion that contribute to TG deposition in the liver [25]. Insulin potently suppresses lipolysis in adipose tissue. However, in a condition involving IR, such as NAFLD, this suppression is frustrated, resulting in an increased efflux of FFAs from adipose tissue. The hyperinsulinemia associated with IR leads to the up-regulation of sterol regulatory element-binding protein-1c (SREBP-1c), which is a key transcriptional regulator of the genes involved in *de novo* lipogenesis and to the inhibition of β-oxidation of FFAs, resulting in the stimulation of lipid accumulation in the liver [26] (Figure 3). Small fat vacuoles (liposomes) around the nucleus (microvesicular fatty change) are the earlier modification: liver cells are filled with multiple fat droplets that do not displace the centrally located nucleus. Thereafter, an incremental size increase of the vacuoles occurs and the nucleus is pushed to the periphery with the characteristic signet ring appearance (macrovesicular fatty change). These well-defined vesicles are optically “empty” because fats vanish during histology processing. Coalescence of large vacuoles into fatty cysts leads to irreversible lesions. Macrovesicular steatosis is the most common form and is associated with alcohol, diabetes, obesity and corticosteroids. There are two discrete forms of steatosis that may be found in patients infected with HCV. Metabolic steatosis can coexist with HCV, regardless of genotype, in patients with risk factors such as obesity, hyperlipidemia and insulin resistance. The second form of hepatic steatosis in HCV patients is a result of the direct cytopathic effect of genotype 3 viral infections; it is a macrovesicular steatosis directly related to the burden of the HCV RNA load and distributed in the periportal areas rather than the centrilobular region which is more commonly seen in NAFLD [27].

A special case is focal fatty liver which is a liver area of localized or patchy imaging, usually by ultrasound, of lipid accumulation. Pathogenesis is not always clear and it is seemingly a condition different from NAFLD and NASH which are widespread and quite uniform conditions, despite that it may result from altered venous flow to liver, tissue hypoxia and malabsorption of lipoproteins. The condition has been increasingly detected by abdominal imaging studies and is most commonly occurring subjacent to the liver capsule. We can face a focal fat deposition within an otherwise normal liver or diffuse fat deposition with focal sparing. Focal fat sparing in particular may be confused with liver metastases, and a fine needle biopsy is sometimes performed to differentiate it from malignancy. Our view is different. Despite that the appearance of focal areas of decreased echogenicity in a fatty liver has previously, and currently, been interpreted solely on the basis of their contrast with the surrounding tissue, so that segmentally or subsegmentally distributed hyperechoic areas have been attributed to focal accumulations of fat in an otherwise normal liver, an accurate biopsy study reported carefully that hypoechoic focal lesions within a “bright” steatotic liver are not areas that have been spared by the process of fatty infiltration and, conversely, that focal areas of increased echogenicity should not be considered to reflect localized accumulations of fat surrounded by normal parenchyma. The difference between these lesions and the hepatic parenchyma surrounding them appears to lie in the organization of this fat into lipid droplets. So, organization of fat into a large number of small droplets would thus be expected to produce a relatively hyperechoic image, although the same quantity of fat divided into a few larger droplets should be reflected in relatively hypoechoic imaging. Focal defects are likely to be areas with different patterns of fatty infiltration as compared with the surrounding tissue, their echogenicity depending on the adjustment of the time-gain compensation curve of the scanner; this may also explain the extreme diversity with which these lesions have been described. The reasons for these irregularities in the organization of the fatty deposits are still unclear. Regional differences in blood flow may play an important role in the intrahepatic distribution of fat, but it is also possible that these differences are a result, rather than a cause, of fat deposition, which leads to elevations in portal vein pressure [28]. To understand the NAFLD pathophysiology, numerous animal models (nutritional and genetic) were considered. Data obtained from animal experiments are quite homogenous in contrast with the heterogeneity of the human population. The high inter-individual variability concerns genetic background, comorbidities, physical activity, drugs, diet, lifestyle, etc. Thus, the translatability of the data to humans is limited. The use of dietary models of NAFLD is more relevant than genetic models because few NAFLD patients exhibit evident genetic defects or specific profiles. Most studied are the models with increased lipid import or synthesis in the liver (high-fat diets, high-fructose/sucrose diets, combined diets) or reduced lipid export or catabolism (methionine- and choline-deficient diet; choline-deficient, *L*-amino acid-defined diet). Since NAFLD has a polygenic genetic background, monogenic mouse models do not fully mirror human NAFLD, but they may provide some information concerning particular events in the pathophysiology of NAFLD.

#### Steatohepatitis and fibrosis

Hepatocytes are not the only type of liver cells contributing to steatosis; actually, there is an intensive exchange of molecules among endothelial cells, fibroblasts, cholangiocytes, stellate cells, Kupffer cells and other types of cells such as leukocytes. In steatotic liver, sinusoidal space is markedly reduced and altered due to the compression by fat-rich hepatocytes as well as the infiltration of macrophages (Kupffer cells) leading to alteration in endothelial signaling toward leukocyte recruitment and activation. Fatty acids may contribute to pro-inflammatory stimulation of leukocytes or macrophages, leading to liver cell injury and disease progression. Cytokines, particularly TNF-α, IL-1b and interleukin-6 (IL-6), are involved in the recruitment of circulating macrophages into the liver and the activation of Kupffer and hepatic stellate cells (HSCs), both contributing to the progression from simple steatosis to steatohepatitis. In humans, serum levels of TNF-α are significantly higher in patients with simple steatosis and NASH than in normal subjects, and TNF-α levels correlate with the stage of fibrosis and with the NAFLD activity score (NAS) in NASH [29]. Activated Kupffer cells, infiltrating monocytes, activated and aggregated platelets, and damaged hepatocytes are the sources of platelet-derived growth factor (PDGF) and tumour growth factor beta-1 (TGF-β1), which trigger the initiation of intracellular signaling cascades that stimulate the proliferation and phenotypic transformation of HSCs into myofibroblast-like cells (MFB), producing collagen [30]. Simultaneously with steatohepatitis, hepatic fibrosis may occur. It is characterized by the overproduction of extracellular matrix proteins, such as collagen type I and III, and preferred deposition in the subendothelial space of Disse leading to the formation of an incomplete subendothelial basement membrane, creating additional diffusion barriers between hepatocytes and the liver sinusoid (“capillarization of sinusoids”) [31]. The development of MFB is the result of a multistep sequence, which originates from liver cell necrosis induced by various noxious agents (toxic, immunologic). Stellate cells are also activated by long-chain polyunsaturated fatty acid [31] and by hormones; the rennin-angiotensin-aldosterone system, for example, plays a role in the fibrosis onset [32]. TGF-β1 is one of the cytokines primarily responsible for these effects and for decreased degradation of matrix proteins [33]. In NAFLD, oxidative stress inhibits the replication of mature hepatocytes, leading to the expansion of the hepatic progenitor cell (oval cell) population. The number of oval cells is reported to correlate with the stage of fibrosis in patients with both alcoholic fatty liver disease and NAFLD. Thus, excessive hepatocyte cell death and inadequate oval cell proliferation are thought to be involved in NASH fibrogenesis [34]. Experimental studies suggest that uncontrolled hepatocyte apoptosis may be a central mechanism triggering liver fibrogenesis and fibrosis [35]. DNA from apoptotic hepatocytes acts as an important mediator of HSC differentiation by providing a stop signal to mobile HSCs when they have reached an area of apoptosing hepatocytes and inducing a stationary phenotype-associated up-regulation of collagen production [36] (Figure 3).

#### Diagnosis

Steatohepatitis is characterized microscopically by steatosis with mixed lobular inflammation, ballooning degeneration of hepatocytes (sometimes with identifiable Mallory bodies), glycogenated hepatocyte nuclei and pericellular fibrosis. There is also a “chicken wire” pattern of pericellular fibrosis, which affects portal areas only secondarily in later stages.

##### Clinical assessment

**Figure 4:**
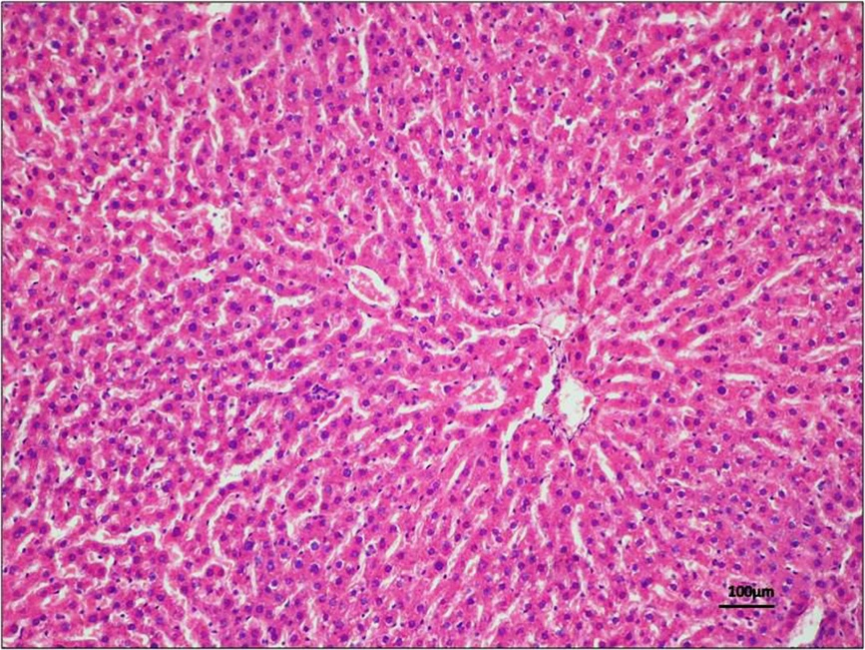
**Morphological and histological analysis of a liver sample.**Haematoxylin and eosin (H&E) staining in liver tissue demonstrated no sign of tissue degeneration, with a good preservation and absence of lipid droplets. NAFLD activity score = 0 for every histological feature. Magnification × 20; scale bar: 100 μm.

**Figure 5:**
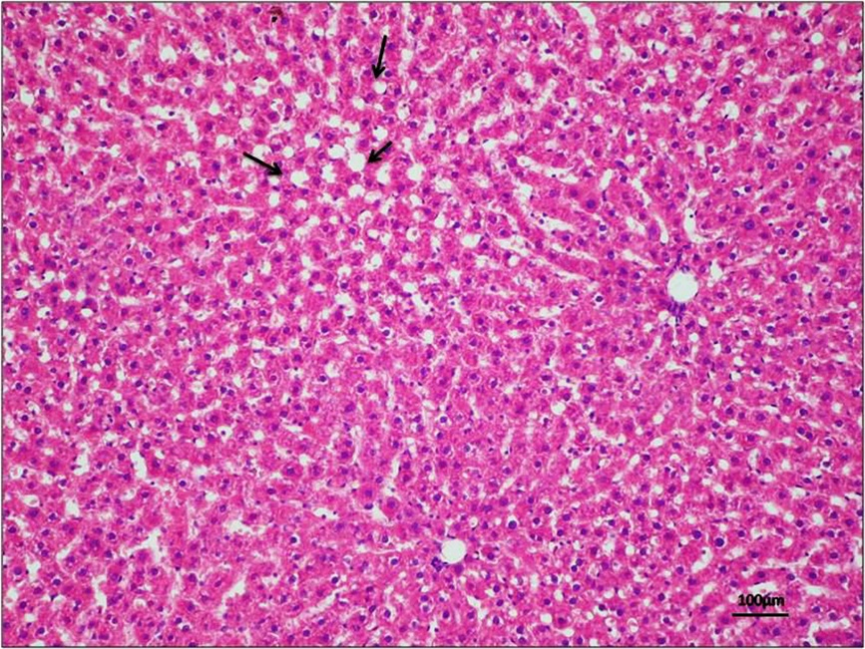
**Morphological and histological analysis of a liver sample.** H&E staining in liver tissue demonstrated no sign of tissue degeneration, with a good preservation and the beginning of formation of lipid droplets (black arrows). NAFLD activity score = 1 for steatosis, 0 for lobular inflammation, 1 for hepatocellular ballooning and 0 for fibrosis. Magnification × 20; scale bar: 100 μm.

NAFLD is an asymptomatic disease; usually, people do not know that their liver is damaged by the presence of fat, even when inflammation is present. Some patients report fatigue or malaise and a sensation of fullness or discomfort in the right hypochondrium i.e. a suboptimal health status. In most cases, hepatomegaly is the only physical sign. Anthropometric indicators of regional adiposity including waist circumference (WC) that correlates with visceral fat (VF), body mass index (BMI), expression of total body fat and waist-to-hip ratio (WHR) are associated to liver injury and fibrosis in NAFLD. WHR is the most important anthropometric indicator for the prediction of NAFLD and independent of other risk factors for metabolic syndrome. Moreover, other anthropometric measures are also closely associated with the occurrence of NAFLD [37]. This indicates that central obesity has a strong relation with liver steatosis. There are some gender differences: in fact, unlike in males, the degree of fat deposition in the liver correlated well with VF area at the umbilicus in females, but WC had no correlation with lipid liver content. One explanation may be that probably in females, not only visceral but also subcutaneous adipose tissue (SAT) contributes to WC at the umbilical level [38]. Moreover, in another study, men and women with lower arm fat depots and women with bigger WC have a greater likelihood of liver injury. So, simple anthropometric measurements of peripheral fat deposits may help stratify liver disease risk [39]. Laboratory abnormalities may be also found; serum levels of aspartate aminotransferase (AST) and ALT could be mildly to moderately elevated. The ratio AST/ALT is usually less than 1 and it could be a useful tool to distinguish NAFLD from steatosis of alcoholic origin, where often the ratio is 2 or more [40]. Unfortunately, the ratio increases as fibrosis advances, losing its accuracy in patients with non-alcoholic cirrhosis [41]. Serum alkaline phosphatase, γ-glutamyltransferase, or both are over the normal range in many patients, although the levels usually are less than those found in patients with alcoholic hepatitis. When the disease progress to the cirrhotic stage, hypoalbuminemia, prolonged prothrombin time, hyperbilirubinemia and diminished platelet count are common. Although pathologic diagnosis through liver biopsy remains the gold standard for the diagnosis, radiological non-invasive scans are widely used. Biopsy specimens may be not adequate and representative of the entire liver, with a high degree of sampling variability, significant intra- and inter-observer variability in histological staging and has a small, but real, risk of complications (mainly bleeding), patient’s discomfort or pain. NAS is a histologic scoring system developed and validated by the Pathology Committee of the NASH Clinical Research Network, which addresses the full spectrum of lesions of NAFLD. The scoring system comprised 14 histological features, 4 of which are evaluated semi-quantitatively: steatosis (0–3), lobular inflammation (0–3), hepatocellular ballooning (0–2) and fibrosis (0–4). Another nine features are recorded as present or absent. A NAS of 5 or greater is consistent with NASH, while a score of less than 3 can exclude this diagnosis [42] (Table 1; Figures 4 and 5).

**Table 1:** **The NAFLD activity score (NAS)**

Histological features	Description	Score
Steatosis (%)	≤5	0
	5–33	1
	33–66	2
	≥66	3
Lobular inflammation	No	0
	≤2 foci	1
	2–4 foci	2
	≥4 foci	3
Hepatocellular ballooning	No	0
	Moderate ballooning	1
	Evident ballooning	2

It provides a numerical score for patients with NASH. NAS is the sum of the separate scores for steatosis (0–3), lobular inflammation (0–3) and hepatocellular ballooning (0–2). The majority of patients with NASH have a NAS score of ≥5.

##### Radiological tools

**Figure 6:**
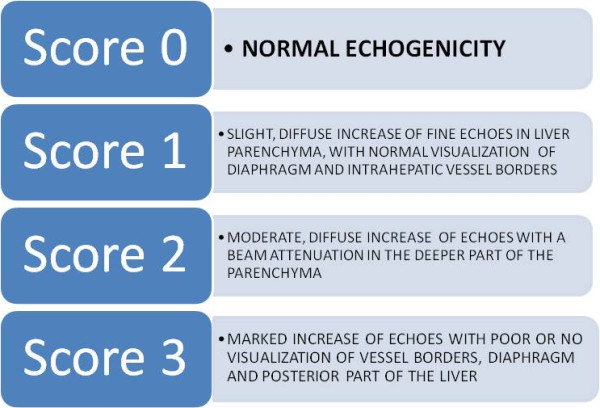
**Bright liver score.** The severity of steatosis can be evaluated by ultrasonography as fatty infiltration in the liver produces a diffuse increase in echogenicity.

Ultrasound (US) is a low-cost, prompt and non-invasive tool to make diagnosis of NAFLD. Fatty infiltration produces a diffuse increase in echogenicity as compared with that of the cortical part of the right kidney (bright liver). Several authors use a score (bright liver score, BLS) to grade the severity of steatosis [43] (Figure 6). US assessment is operator dependent, so that the inter-observer variability is a drawback. On computed tomography (CT) scan, the presence of fat leads a low-density hepatic parenchyma. Steatosis involves all parenchyma in a large amount of patients, but in some cases, the disease is not diffuse but focal. In this situation, US and CT could misinterpret as showing masses of uncertain significance or actual malignancy. In these cases, contrast-enhanced ultrasound (CEUS) or magnetic resonance imaging (MRI) can differentiate easily space-occupying lesions from focal fatty infiltration or focal fatty sparing (isolated areas of normal liver in the context of steatosis). CEUS uses microbubble to enhance and improve the US imaging; it can be used as a kinetic tracer to assess the severity of liver disease, underlining the local and systemic hemodynamic changes that accompany chronic liver disease [44]. “Transit time” curves are generated by tracking the passage of a peripherally administered intravenous microbubble bolus through the circulation [45, 46]. These times have not yet been validated in NAFLD but even if some studies of the parenchymal phase of contrast enhancement have shown decreased signal intensity in NASH patients, this is only a conjectural insight for the detection of NASH [47]. MRI may quantify hepatic steatosis based on the signal differences between fat and water. Limitation of this scan include costs, inability to use it in patients with implantable devices or those with claustrophobia, and altered values in patients with iron overload [48]. Proton magnetic resonance spectroscopy (^1^H-MRS) gives a quantitative assessment of fatty infiltration of the liver. It is an effective, non-invasive technique that can be used to diagnose and quantify hepatic steatosis corresponding with histopathologic grades [49]. A recent meta-analysis showed that MRI and ^1^H-MRS have more accuracy than US and CT in the diagnosis of NAFLD. These findings suggest that MRI and ^1^H-MRS also perform better than US and CT for detecting separate disease grades, especially for mild disease (<30% steatosis). This is of great importance in clinical practice when an accurate estimation of the severity of hepatic steatosis is needed. Additional benefits of MRI and ^1^H-MRS over US are the quantitative measurements which have minor subject to inter- and intra-observer variability. Disadvantages of CT are the radiation exposure and factors affecting the accuracy of the results, such as imaging parameters or iron accumulation [50]. The severity of hepatic steatosis can be accurately determined radiologically only when there is a moderate or severe (>33%) fatty infiltration of the liver documented by a liver biopsy. Moreover, despite the excellent sensitivity in detecting significant steatosis, radiological modalities are unable to distinguish between NAFLD and NASH [51]. There are, however, imaging techniques that can assess fibrosis non-invasively such as transient elastography (FibroScan) and acoustic radiation force impulse (ARFI), which use the US waves to measure the liver stiffness, providing also a graduation. Also, magnetic resonance elastography (MRE) uses the differing viscoelastic properties of healthy and disease livers, being an MR-based technique. FibroScan consists of an US transducer that cannot visualize any image but can assess the elasticity of the tissue measuring the velocity of the waves; a limitation is the difficulty in accurately assessing obese patients, frequently affected by NAFLD. A new XL probe was used for this kind of patients with less frequent failure of measurement; discordance between liver fibrosis estimated by biopsy and TE using the FibroScan XL probe was infrequent, but patients with severe obesity and elevated liver stiffness have the greatest risk of discordance [52]. It is reported that the velocity of elastic waves is faster in fibrotic liver than in normal liver. The mean cutoff for the diagnosis of significant fibrosis and cirrhosis were 0.84 and 0.94, respectively, so that FibroScan is rather accurate for the diagnosis of high grades of fibrosis [53]. Moreover, the controlled attenuation parameter (CAP), evaluated with transient elastography, could efficiently evaluate steatosis grades [54]. ARFI is, differently, an US-based elastography method that is integrated in a conventional US machine enabling the exact localization of measurement site. A recent study reported no significant difference in diagnostic accuracy for the non-invasive assessment of liver fibrosis between transient elastography and ARFI [55].

#### Serum markers

Several diagnostic panels have been proposed to predict steatosis and fibrosis based on serum measurements. SteatoTest (ST) and NashTest (NT) would permit a simple and non-invasive semi-quantitative estimate of steatosis and NASH combining 13 parameters: age, sex, height, weight, triglycerides, cholesterol, alpha-2-macroglobulin, apolipoprotein A1, haptoglobin, gamma-glutamyltranspeptidase, transaminases ALT, AST and total bilirubin [56], but their reliability is not demonstrated. NAFLD liver fat score was derived from a Finnish population as useful tool in identifying patients who may be at risk of developing non-alcoholic steatohepatitis and as help to address the selection of the appropriate anti-hyperglycemic drugs for type 2 diabetic patients. The score incorporates simple variables such as the presence of metabolic syndrome and type 2 diabetes mellitus, fasting serum insulin, AST level and AST/ALT ratio [57]. FibroTest (FT) is the most frequently used serum fibrosis marker and consists of an algorithm of five fibrosis markers (alpha-2-macroglobulin, apolipoprotein A1, haptoglobin, GT, bilirubin). The enhanced liver fibrosis (ELF) test consists of an algorithm of three fibrosis markers (hyaluronic acid, amino-terminal pro-peptide of type III collagen, tissue inhibitor of matrix metalloproteinase-1). It was claimed that FibroTest and ELF can be performed with comparable diagnostic accuracy for non-invasive staging of liver fibrosis [58], but their actual clinical validation is not achieved nor confirmed. The AST/platelet ratio index (APRI) is another good estimator of hepatic fibrosis [59], whose reliability is not demonstrated. Another group of researchers developed the NAFLD fibrosis scoring system using six commonly measured parameters that are independent indicators of advanced liver fibrosis (age, hyperglycemia, BMI, platelet count, albumin and AST/ALT ratio). They reported that this method could easily separate patients with NAFLD with and without advanced fibrosis, so that liver biopsy for identification of advanced fibrosis would be unnecessary in a substantial proportion of patients (75% or more). The NAFLD fibrosis score is an example of an indirect panel marker, which includes clinical, anthropometric and blood derived data, and can reliably exclude advanced fibrosis with a high negative predictive value (NPV) [60]; nonetheless, this approach is not sufficiently validated and not widely used and accepted. Serum biomarkers produced during fibrogenesis were investigate to predict the presence of fibrosis. Rosenberg et al. made an algorithm combining age, hyaluronic acid, amino-terminal pro-peptide of type III collagen and tissue inhibitor of matrix metalloproteinase-1 with high sensitivity and specificity [61]. Another field of interest has been the measurement of adiponectin in NAFLD patients who have markedly lower plasma concentrations of this adipokine than control subjects. Furthermore, low adiponectin levels are strongly associated with the severity of liver histology including steatosis, necroinflammation and fibrosis [62]. Hepatic apoptosis is an essential feature of NASH, so hepatic biomarkers of apoptosis were also investigated. Plasma levels of caspase-generated cytokeratin 18 (CK-18), a protein involved in the apoptosis cascade, correlate with the amount of hepatocyte apoptosis and independently predict the presence of NASH [63]. A study on NAFLD patients without a diagnosis of MS showed that the haemoglobin level was the only variable independently associated with NASH and fibrosis (>2). In this study, a cutoff of 147 g/L of haemoglobin was proposed as predictor of advanced fibrosis, claiming high sensitivity and specificity. The main hypothesis was that because haemoglobin concentrations increased similarly with the increase of blood viscosity, determining an increased peripheral resistance and reduced hepatic perfusion, this behaviour has been suggested to accelerate fibrosis. Furthermore, increased iron storage in the liver could damage it through oxidative stress and lipid peroxidation [64]; also, this approach, which appears quite conjectural and unpractical, is not widely accepted. The microbial community of the intestine consists of more than 500 species and many of them have not been identified so far. The intestinal microflora has many important functions, in particular to maintain the microbial barrier against established as well as potential pathogens, and furthermore, it influences the motility and perfusion of the intestinal wall and stimulates the intestinal immune system, reducing bacterial translocation and producing vitamins [65]. It has been reported that both in animal and human studies, obese individuals have an aberrant and peculiar intestinal microbiota composition, which appears to be linked to the obese state itself and yet susceptible to dietary modulation and can impact on weight loss in humans [66]. NAFLD and NASH have been associated with small intestinal bacterial overgrowth (SIBO) and increase intestinal permeability in patients who underwent intestinal bypass. In animal models with SIBO and confirmed NASH histology, the disease reverts after antibiotic therapy. Several bacterial bioproducts may be potentially hepatotoxic; the most involved in the pathogenesis of NAFLD is a lipopolysaccharide endotoxin, an element of the cell wall of Gram-negative bacteria, absorbed during lipid absorption. It can stimulate inflammatory pathway and promote IR, hepatic steatosis and fibrosis [67]. Studies have confirmed that intestinal permeability and SIBO are increased in NAFLD patients and that these factors are associated with the severity of hepatic steatosis [68]. In normal circumstances, only small amounts of endotoxin will cross from the intestinal lumen into the systemic circulation and the absorbed endotoxin will rapidly be removed by monocytes, particularly Kupffer cells that are residents in the liver. Patients with NAFLD are characterized by a significant increase in circulating levels of endotoxin. Although no significant difference in endotoxin levels was observed between patients with simple steatosis (NAFLD) and patients with NASH, endotoxin levels may represent an important, even not established, early marker of potential liver abnormality [69]. A comprehensive analysis of plasma lipids and eicosanoid metabolites, quantified by mass spectrometry, in NAFLD patients showed significantly increased total plasma monounsaturated fatty acids and the increased ratio of monounsaturated fatty acids/unsaturated fatty acid with variations in polyunsaturated fatty acids indicating that NAFLD is associated with *de novo* lipogenesis. Furthermore, eicosanoids and products of oxidation of the inflammatory arachidonic acid by lipoxygenase are particularly affected during progression to NAFLD and then NASH [70]. In brief, there is not yet any single tool or algorithm sufficiently and practically reliable to diagnose and differentiate NAFLD from NASH, whose diagnosis still relies on a combination of serum and imaging features.

#### New perspectives

The concept of interactome can also mean indirect interactions among genes, i.e. genetic interactions, environment and personal behaviour, and this is the frame of the present review. This can include many aspects and approach, which were used and challenged also in NAFLD. Metabonomics is the study of metabolism and metabolic response to stimuli; it is usually achieved through the statistical analysis of the spectra obtained by nuclear magnetic resonance spectroscopy and mass spectrometry [71]. ^1^H NMR spectroscopy is used for identification of structures in biofluids (cell media, urine, plasma), intact biopsy specimens and tissues. Metabolomic strategies have been used to study the pathogenic mechanism of metabolic syndrome, NAFLD and NASH. Several metabolites derived from human and gut microbial co-metabolism have been associated with phenotypes of metabolic syndrome. Genetic factors determine metabolic phenotypes, and metabotypes can be treated like any other phenotype in a genetic analysis, which means that variations of each metabotype arise from genetic and environmental factors. The gut microbiome has a strong effect on host metabolic phenotypes, which are relayed by complex signaling pathways that connect organs such as the liver, brain and immune system [71]. Methylamines are a class of metabolites produced by the intestinal microbiota and were identified as markers of NAFLD in mice. Metabolic profiling technologies could be used in a clinical setting for epidemiological studies, prognosis assessment and population stratification [71]. Moreover, micro-RNAs (miRNAs) are an emerging class of highly stable, non-coding small RNAs that regulate gene expression at the post-transcriptional level. Deregulation of miRNA expression may be a key pathogenic factor in many liver diseases. Dumas et al. demonstrate a serum miRNA panel with considerable clinical value for the diagnosis of NAFLD [72].

In Germany, a national flagship research programme called Virtual Liver Network (VLN) focuses on producing a validated computer model of human liver physiology in order to improve the understanding of the dynamics and the complex functions and applying this to support clinical practice. Given its role in drug metabolism, these models could be used in the future to analyse patient-derived data and optimize drug efficacy and patient safety [73].

Other authors developed a mathematical model of the hepatic lipid dynamics to simulate the fate of fatty acids in hepatocytes. According to this model, steatosis development is based on the processes of hepatocyte swelling and impaired hepatic microcirculation with a fundamental role of oxygen availability for lipid oxidation [74]. All these techniques need future validations to be used in the daily clinical practice but represent new interesting insights in the study of the liver.

#### Suggestions for improving diagnostic reliability

In the daily practice, we suggest to exert greater efforts than we do currently to better understand the clinical frame of any individual patient. Reliable information on the quality of diet and lifestyle are mandatory for predicting and preventing this liver disease which is associated with unhealthy dietary habits and sedentary life and, at last, obesity. This is the only sustainable way to address a personalized approach to management, which includes diagnostic tools and the necessary follow-up and therapeutic strategies. The fourth P, “participatory”, is the key of this comprehensive approach: if the physician and the patient rely excessively, or exclusively, on laboratory information and on pharmacological agents, the battle against NAFLD is already lost. Non-invasive imaging modalities are still the best surrogate of liver biopsy, which is not warranted, unless the need of investigating a different condition, and particularly cancer, is present. Since also the outcome of the current pharmacological approaches is substantially disappointing, personalization of medicine by participation is not only the therapeutic step, but, much more, the diagnostic stage, which includes a proactive patient-doctor relationship, with the old-fashioned components of history, physical examination and collateral information acquisition.

#### Prevention and treatment

##### Diet

Like biomarkers, studied to diagnose NASH non-invasively, current therapies are focused on the various pathways thought central in the pathogenesis of this disease. Often, patients with NAFLD are obese or overweight and insulin resistant with higher energy intake with respect to individuals without hepatic steatosis [75]. In some nutritional reports, it has been suggested that high-fat, high-fat plus low-protein, high-carbohydrate and/or high-cholesterol diets are the main causes of NAFLD. Although many NAFLD patients show excess caloric intake, obesity and/or IR, not all patients present these features. In a nutritional analysis on Japanese population, non-obese NAFLD patients had some features that differed from those of obese patients [76]. The dietary levels of total calories, fat and carbohydrate were significantly higher in obese NAFLD patients with IR than those in non-obese NAFLD patients and healthy volunteers. The most interesting finding was that cholesterol intake was markedly higher in non-obese NAFLD patients than in obese NAFLD patients, although cholesterol intake in obese patients was also significantly higher than that in healthy volunteers. Moreover, serum cholesterol levels may be normal in NAFLD patients because dietary cholesterol is promptly taken up into the hepatocyte cholesterol pool [76]. Fatty liver without obesity can, indeed, be established in animal models by feeding them with a hypercholesterolemic diet containing normal calorie level [77]. Another study showed that mice treated with trans fats in a high-fat diet and high-fructose corn syrup in water, associated to sedentary behaviour, developed a severe phenotype of fatty liver disease with necroinflammatory changes and a profibrogenic response at the molecular level in the setting of obesity, hepatic IR, impaired insulin responsiveness and hyperinsulinemia. The presence of trans fats promoted fat retention in the liver and contributed substantially to hepatocellular injury; moreover, fructose is commonly found in soft drinks and other carbohydrate-sweetened beverages, the favourite drink of Western diet [78]. Fructose was originally promoted as a beneficial dietary component because it does not stimulate insulin secretion. But since insulin signaling plays an important role in central mechanisms of satiety, this property of fructose may be undesirable [78]. Furthermore, the consumption of sucrose-sweetened soft drinks with a daily intake of 1 L results in a higher relative amount of VAT and liver fat accumulation than isocaloric semi-skimmed milk, aspartame-sweetened beverages and water [79]. A study comparing fructose-sweetened and glucose-sweetened beverages revealed that mainly fructose-containing drinks increase visceral adiposity and lipids in overweight subjects [80]. The same authors reported that subjects with MS consuming fructose-sweetened beverages exhibited the largest decreases of postprandial fat oxidation rates and increases of carbohydrate oxidation rates.

Moreover, resting energy expenditure is reduced compared to baseline values in subjects consuming fructose-sweetened beverages [81]. Treatment of all metabolic-associated conditions is crucial in NAFLD therapy. Lifestyle modifications are effective in reducing intrahepatic triacylglycerol concentration and circulating liver enzymes and improving measures of glucose control and/or insulin sensitivity in patients with NAFLD. A study demonstrated that changes in dietary fat content can modify liver fat within 2 weeks. The diet did not change body weight, FFA concentrations and visceral or subcutaneous fat mass, but liver fat decreased during the low-fat diet and increased during the high-fat diet. The changes in liver fat were paralleled by changes in fasting serum insulin concentrations [82].

**Figure 7:**
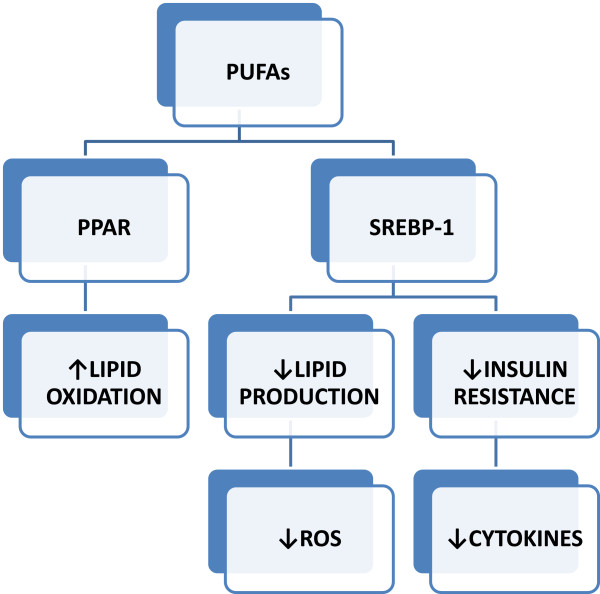
**PUFAs molecular mechanism of action.** ω-3 PUFAs regulate gene transcription factors, i.e. peroxisome proliferator-activated receptor alpha (PPARα), PPARγ and sterol regulatory element-binding protein-1 (SREBP-1), having a beneficial impact on most of the cardiometabolic risk factors (hypertension, hyperlipidemia, endothelial dysfunction and atherosclerosis). Activation of PPARs stimulates lipid oxidation, decreases the endogenous lipid production and determines IR, besides a significant reduction of the expression of pro-inflammatory molecules (TNF-α and IL-6) and of oxygen reactive species, leading to amelioration of hepatic steatosis.

The use of omega-3 polyunsaturated fatty acids (ω-3 PUFAs) has been described as a potential treatment of NAFLD [83] (Figure 7). ω-9 oleic acid is the most prevalent monounsaturated fatty acid (MUFA) in the diet, and olive oil is one of its major sources (other sources are nuts and avocado). Olive oil has traditionally been the principal oil of the Mediterranean diet [84]. The beneficial effect of MUFAs on the risk of cardiovascular disease and on lipid profile has been studied. Dietary oleic acid decreased oxidized LDL, LDL cholesterol and TG concentration without alterations in HDL levels. It has been demonstrated that consumption of MUFAs decreases blood TGs by increasing fatty acid oxidation similarly to PUFAs through activation of PPARα and PPARγ and by reducing the activation of SREBP-1. This causes inhibition of lipogenesis, increase of lipid oxidation and decrease of IR, leading to a reduction in hepatic steatosis [85]. MUFA diet prevents central body fat accumulation and decreases postprandial adiponectin expression induced by a carbohydrate-rich diet in insulin-resistant subjects [86]. Additional effects of olive oil beyond its MUFA composition relate to its polyphenols. Polyphenols present in olive oil, such as oleuropein, hydroxytyrosol, tyrosol and caffeic acid, have an important antioxidant and anti-inflammatory effect [85, 87]. Among polyphenols, resveratrol, a component of several grape species, appears to be particularly relevant in the context of liver disease. Resveratrol has anti-inflammatory effects inducing a decrease in pro-inflammatory cytokines. Its administration reduces portal pressure, hepatic stellate cell activation and liver fibrosis, and improves hepatic endothelial dysfunction in cirrhotic rats [88]. In an interesting study, resveratrol reduced liver weight and TG content and does not modify the activity of lipogenic enzymes but reduces non-esterified fatty acids and alkaline phosphatase as well as liver oxidative stress. This study demonstrates that resveratrol can protect the liver from NAFLD by reducing fatty acid availability and oxidative stress [89]. Another study reports that hepatic steatosis was significantly improved and associated to a decreased TNF-α production in rats treated with resveratrol. Authors think that the decreased liver damage in a model of liver steatosis could be related to its anti-TNF-α effect [90]. It may be a promising candidate treatment for NAFLD and NASH. Although alcohol abuse is detrimental for liver, modest wine consumption is associated with reduced prevalence of suspected NAFLD. A recent study supports the safety of one glass of wine per day for cardioprotection in patients at risk for both coronary heart disease and NAFLD [91]. Another research suggests that light to moderate alcohol consumption may have a protection effect against IR in severely obese patients and it had no impact on the severity of activity and stage of liver disease [92]. Studies have shown the role of soy in the diet and its relation with NAFLD. Indeed, interactions between soy protein, the isoflavonoid-enriched fraction and amino acid pattern may reduce lipogenesis. These soy components modulate lipid and carbohydrate metabolism in the liver through the expression of related transcription factors. One of the possible effects of soy is, in fact, related to its ability to stimulate PPARα and inhibit SREBP-1. Soy and its components might also increase peripheral insulin sensitivity by reducing oxidative stress and modulating pro-inflammatory cytokines [93]. In an animal model, soy protein significantly lowered plasma cholesterol concentrations and body fat accumulation; it decreased also the hepatic lipid depots of triacylglycerols and cholesterol and the concentrations of lipid peroxide. Furthermore, in an analysis of antioxidative status, rats fed with a soy protein diet showed improved antioxidative potential due to increases in superoxide dismutase and catalase activities [94] confirming the protective role on liver damage.

Another putatively promising substance is taurine, naturally present in food, especially in seafood and meat. Taurine reduced or prevented hepatic steatosis in high-sucrose diet-fed rats. Although the mechanisms by which taurine exerts its beneficial effects are unknown, several possibilities were proposed. Substantial evidence indicates that taurine protects a wide variety of cells from oxidative damage by increasing antioxidant defense systems, decreasing the formation of ROS and interfering with ROS activity. Dietary supplementation with taurine offers significant potential as a preventative treatment for NAFLD [95]. Coffee is one of the most popular beverages in the Western world. A recent study showed that consumption of regular coffee was associated with a lower level of fibrosis and was independently protective against fibrosis in a cohort of severely obese patients undergoing bariatric surgery. Moreover, total consumption of coffee in different form, regular coffee and espresso, was not associated with NASH [96]. The methylxanthine caffeine, a main component of coffee, may inhibit the synthesis of connective tissue growth factor in liver parenchymal cells. The gene expression in the liver and adipose tissues of mice that were fed by a high-fat diet with added coffee was investigated; in this model, a strong induction of anti-inflammatory responses was found with lower levels of inflammatory cytokines (i.e. TNF-α, IL-6) resulting in an improvement of fatty liver [97]. In human, coffee drinking in patients with NAFLD is inversely associated with the degree of bright liver appearance at US, with a trend opposite to overweightness. Authors hypothesized a possible favourable and/or preventive role of coffee use in the natural history of NAFLD [98]. Choline is a water-soluble essential nutrient usually grouped within the B-complex vitamins. It is found in meat, fish and egg but also in vegetables such as cauliflower. Choline influences liver function, and the dietary requirement for this nutrient varies depending on an individual’s genotype and estrogen status. It is unknown why some individuals develop NAFLD and others do not and why some patients tolerate total parenteral nutrition and others develop liver dysfunction. Gut microbiota may influence bioavailability of dietary choline to the host and thereby contribute to the development of NAFLD [99]. The role of vitamin D in the pathogenesis of hepatic diseases is actually of great interest. In the liver, vitamin D suppresses fibroblast proliferation and collagen production. A strong independent association was found between low 25 (OH) vitamin D levels and NAFLD; this association is independent from diabetes, lipid profile alterations and IR. Besides, an inverse correlation between serum 25 (OH) vitamin D levels and the degree of NAFLD was observed, suggesting that vitamin D may exert a dose-dependent effect on fat accumulation into the hepatocytes [100]. In Table 2, we summarize several possible dietary supplements, with their respective effects in NAFLD patients. Despite all these studies, and many others are available, the road of the search for the benefits, or the disadvantage, of single nutrients, managed as they were drugs, is substantially disappointing, and more realistic approaches address the assessment and the change of nutritional profiles, whose healthy features are defined by epidemiology and by some clinical and experimental evidence [43].

**Table 2:** **Dietary supplements in NAFLD treatment**

Dietary supplements	Effects
PUFAs/MUFAs	• Increase of fatty acid oxidation
	• Decrease of endogenous lipid production and insulin resistance
Polyphenols (oleuropein, hydroxytyrosol, tyrosol, caffeic acid, resveratrol)	• Antioxidant and anti-inflammatory effects
Soy proteins	• Modulation of lipid and carbohydrate metabolism
	• Reduction of oxidative stress
Taurine	• Increase of antioxidant defense
Coffee	• Protection against fibrosis
	• Anti-inflammatory effects
Choline	• Influences liver function
Vitamin D	• Suppression of fibroblast proliferation and collagen production
	• Effect on fat accumulation into the hepatocytes

List of the dietary supplements, in which the effects were evaluated in the treatment/therapy of non-alcoholic fatty liver disease.

##### Lifestyle

The current target of intervention is addressed to improve health and quality of life by lifestyle changes [101]. Lifestyle was originally introduced as a special derivative of style in fashion and art. Nowadays, there is a comprehensive frame of the factors interacting for maintaining physical and mental health. These lifestyle factors are deemed as crucial to an individual’s longevity and to prevent disease, such as obesity and NAFLD. Few aspects of lifestyle are voluntary, and fashion and marketing are significant factors. Many fashion models may have dual effects on health. The trend of ultra-slim models has been limited from the runways for a while, but now they are on the stage; these are effective and beneficial icons of the fight against obesity throughout the world. There is a relevant social “side effect” on mental anorexia occurrence, which is quite questionable and, probably, very limited. Also, unfavorable effects of the opposite model can be detected. The universal availability of comfortable and wide clothing is contributing to the enhancement of obesity. Fashion and health are symbiotic. Fashion can make us stand straighter and help prevent spine pain and osteoporosis. Fashion can damage our feet and our balance through the choice of shoes or it can exercise our leg muscles and improve our gait. Fashion can protect us from the harsh rays of the sun. Now, fashion aims even to monitor our health through textile embedded electronic sensors. High heels can cause poor posture, put pressure on joints and lead to arthritis, hammer toes, back pain and tendon injuries. Low-rise pants, “low-cut jeans”, “hipster jeans” and “lowriders” intended to sit low on or below the hips may put pressure on sensory lateral cutaneous nerve of the thigh, which can cause pain and paresthesia in the nerve’s area of distribution; also, effects on digestion, i.e. esophageal reflux, constipation and others, are frequently observed. Mostly breathable materials like cotton or linen reduce yeast and fungal infections, and for men, wearing tight trousers can cause overheating of the testes, lowering sperm count and causing fertility problems. As researchers attempt to conceptualize relationship among different events, similarly, fashion designers attempt to design clothes which are functional as well as aesthetically pleasing. The concept of functional, however, is not yet comprehensive of healthy. There are still limited or absent researches for a realistic paradigms of healthy clothing, which currently means comfortable according to weather, age and activity. Full interactive and comprehensive analysis of relationship of fashion, lifestyle, nutrition, physical exercise, health and disease is in progress through the clinical investigation of a group of youngsters within 14–35 years. By this approach, we should achieve some information also regarding the relationship between sedentary life, also related to not appropriately suited clothing, obesity and malnutrition, which not necessarily means underweight and other aspects which are strongly related to fashion models and to media.

Despite that this is the actual scenario of lifestyle and its implications cannot be oversimplified without losing meaning and translational prospects, several investigations, also in humans, tried to simulate in experimental subset lifestyle and its changes.

In a very significant study, healthy volunteers ate at least two fast food-based meals a day, led a sedentary lifestyle for 4 weeks, increasing in body weight of 5%–15%, and underwent proton magnetic resonance spectroscopy (^1^H-MRS) to evaluate hepatic triglyceride content (HTGC). This change in lifestyle induced an alteration in ALT levels and a visceral accumulation of adipose tissue as shown by the significant rise of WC; a consistent increase of HTGC also occurred, indicating a net retention of lipids within hepatocytes [102]. Energy restriction, with and without increased physical activity, and weight reduction were the most frequently employed methods to reduce liver fatty infiltration. Weight reductions of 4%–14% resulted in statistically significant relative reductions in reducing intrahepatic triacylglycerol concentration of 35%–81%. Low (800–1,800 kcal/day) and very low-calorie diets (<800 kcal/day) and/or carbohydrate restriction (20–50 g/day) resulted in the most rapid reductions in body weight and NAFLD [103]. Another study showed a significant decline in the aminotransferase levels in patients with NASH who adhered to the aerobic exercise program regardless of their weight loss. All these patients had a persistent elevation of serum aminotransferases prior to commencing exercise, and the fall in elevated liver enzymes was noticed within 3 months of joining the program. Aerobic exercise of sufficient intensity and duration has beneficial effects on insulin sensitivity and alters substrate use in skeletal muscle. Furthermore, the decrease in WC is likely to be due to a decrease in abdominal fat stores. Exercise seems to redistribute the fat stores in the body, which ultimately leads to a decrease in visceral obesity and heightens the insulin responsiveness in adipose tissue [104]. Moreover, IR, WC, BMI and ALT levels correlate with histological improvement of NAFLD [105]. Another study showed an inverse relationship between vigorous-intensity physical activity and NAFLD severity. Moderate-intensity physical activity and total volume of physical activity were not related to outcomes. Thus, intensity may be an important dimension of physical activity to consider when counseling patients and planning interventions [106]. Emerging evidence suggests that obstructive sleep apnea (OSA), the recurrent obstruction of the upper airway during sleep, may play a role in the progression of hepatic steatosis and the development of NASH. OSA results in fragmentation of sleep and in recurrent oxyhaemoglobin desaturations leading to intermittent hypoxia (IH). Recent studies showed that in patients with OSA, the severity of IH predicted the severity of NAFLD on liver biopsy. Mouse model showed that IH causes TG accumulation in the liver and liver injury and hepatic inflammation. Several causes are hypothesized: IH may worsen hepatic steatosis by inducing adipose tissue lipolysis, also through the sympathetic nervous system, and increasing lipid biosynthetic pathways. IH up-regulates hypoxia-inducible factor 1 alpha (HIF-1α) and possibly HIF-2α, which may increase hepatic steatosis and induce liver inflammation and fibrosis. IH may also up-regulate ROS generation via NADPH oxidase system stimulating hepatic stellate leading to liver fibrosis [107]. Although OSA is associated with manifestations of the metabolic syndrome, recent studies in healthy human volunteers revealed that increasing HI is associated with worsening insulin IR independent of obesity [108]. Furthermore, IR and dyslipidemia induced by OSA can be reversed by continuous positive airway pressure. Patients may benefit from identification and treatment for OSA because it may improve liver steatosis [109]. Bariatric surgery is a specific treatment considered in patients who have a BMI greater than 40 kg/m^2^ or with a BMI of 35 kg/m^2^ who have obesity-related comorbidities. It induces weight loss by reducing the size of a patient’s stomach by either removing a portion of the stomach, using a gastric band, or by gastric bypass. Several studies have found that bariatric surgery has also effects on insulin resistance, steatosis and liver inflammation. The role and the validation of this treatment for NAFLD is not clear, and future well-designed studies need to be conducted [110]. Detrimental effects of marketing food information through misleading or actually fake message must be understood, detected and counteracted: in this sense, the active and informed participation of the patient is the key and the premise for the further workup addressed to personalized therapy.

##### Drugs

Use and effectiveness of drugs is very far from being demonstrated by the published studies challenging benefits, if any, of the currently available drugs. Given that IR plays a key role in the pathogenesis of NAFLD, many studies have evaluated the use of insulin sensitizers for the improvement of peripheral insulin sensitivity as a possible treatment for this disease. Glitazones (TZDs) are agonists of PPARγ nuclear receptors, superfamily which regulates the transcription of genes involved in lipid metabolism and plays a role in increasing insulin sensitivity as well as in promoting fatty acid uptake into adipocytes. This class of drugs also promote the differentiation of large, insulin-resistant adipocytes into small, metabolically active, insulin-sensitive adipocytes. TZDs decrease, then FFA influx to the liver, decrease TNFα and increase adiponectin production. One of the most reproducible effects of TZDs is a reduction of aminotransferase levels by 30%–58%. This occurs early on with therapy. This effect is short after drug discontinuation; a return to baseline levels usually occurs within 3 months. Longer treatment (>1 year) does not seem to have additional beneficial effects [111, 112].

An intriguing trial compared the use of pioglitazone and vitamin E for the treatment of non-alcoholic steatohepatitis in adults without diabetes. It showed that vitamin E was superior to placebo and suggested that pioglitazone may also have efficacy with highly significant reductions in steatosis, inflammation and hepatocellular ballooning, as well as with improvements in IR and liver-enzyme levels. Neither agent was associated with a significant improvement in the mean fibrosis score after 96 weeks of treatment. The weight gain among the subjects receiving pioglitazone with an increase of peripheral adipose tissue and a concomitant decrease in VF content, which did not resolve after discontinuation of the drug, also discourages its long-term use [111, 113].

Another insulin sensitizer is metformin. Its use as an anti-diabetic drug is explained by its ability to lower blood glucose by decreasing gluconeogenesis in the liver, stimulating glucose uptake in the muscle and increasing fatty acid oxidation in adipose tissue. Furthermore, it helps reduce body weight in obese patients with and without diabetes and induces a significant reduction in total body fat and VF. Weight loss during metformin treatment has been attributed to decreased net caloric intake, probably through appetite suppression. Several trials have evaluated the liver histology modification together with serum aminotransferase levels and IR showing a marked amelioration in NAFLD patients treated with metformin. In terms of histological improvement, some trials showed significant differences in inflammation, steatosis and fibrosis after treatment with metformin [113, 114]. In contrast to these promising results, some recent open-label studies have found no benefit of metformin treatment in liver steatosis, aminotransferase levels and IR markers compared to lifestyle changes or control untreated group [115]. Ezetimibe, a blood cholesterol-lowering agent, is a NPC1L1-specific inhibitor and selectively blocks 54% of cholesterol absorption from the intestine, but does not inhibit the absorption of fat-soluble vitamins. In a recent study on non-obese NAFLD patients showing excess intake of dietary cholesterol, ezetimibe therapy decreased serum ALT levels and improved steatotic findings on ultrasonography in some patients [116]. Statins (3-hydroxy-3-methylglutaryl-coenzyme A reductase inhibitors) are used worldwide to treat lipid disorders, particularly elevated low-density lipoprotein-cholesterol and substantially reduce cardiovascular events and mortality. Although statins are putatively associated with some adverse events, including elevated hepatic enzymes and liver dysfunction, an elevated serum ALT level at baseline attributable to NAFLD is unlikely to increase the risk of statin-associated elevations in ALT. In clinical studies, simvastatin and atorvastatin were associated with a reduction in hepatic steatosis and may inhibit the progression to NASH. Four years of treatment with atorvastatin combined with vitamins C and E reduced hepatic steatosis by 71% in people with NAFLD at baseline. Taken together with a previous report showing that excess cholesterol consumption may accelerate NAFLD, statins may be promising candidates for the treatment of NAFLD [117, 118]. Since PPARα is highly expressed in the liver and is involved in fatty acid oxidation, fibrates and PPARα agonists increase FFA oxidation in the liver, alter TG synthesis and reduce hepatic synthesis of VLDL. A placebo-controlled trial was performed to evaluate the effect of two classes of pharmacological agents used to treat hypertriglyceridemia: fenofibrate and nicotinic acid. Although intrahepatic TG content was not affected by either treatment, both fenofibrate and niacin reduced plasma TG concentrations by altering VLDL-TG metabolism. However, the mechanism responsible for the change in VLDL-TG concentration is different for each drug; fenofibrate increases plasma VLDL-TG clearance, whereas nicotinic acid decreases VLDL-TG secretion [119]. These findings are strengthened by another study on patients with non-alcoholic fatty liver disease treated with fenofibrate that improved metabolic syndrome and glucose and liver tests. However, its effects on liver histology were minimal. Biopsy after treatment, indeed, revealed a decrease in the grade of hepatocellular ballooning degeneration, but the grade of steatosis, lobular inflammation, fibrosis or non-alcoholic fatty liver disease activity score did not change significantly [120]. The renin-angiotensin system (RAS) plays key roles in the regulation of blood pressure and fluid balance, as well as in the pathogenesis of IR and NAFLD. In addition, inhibition of the RAS may improve the intracellular insulin signaling pathway, offering better control of adipose tissue proliferation and adipokine production. The two main classes of RAS blockers are angiotensin II receptor blockers (ARBs) and angiotensin-converting enzyme inhibitors (ACEIs). ARBs have PPARγ-activating properties at low (telmisartan), medium (irbesartan) and very high concentrations (losartan) [121]. Valsartan prevented the pathological progression of hepatic fibrosis in type 2 diabetic rats, which correlated with reducing TGF-β1 and TNF-α expression and has also anti-apoptosis and mitochondria-protective potential [122]. Losartan significantly decreased the steatosis degree and the SAT and VAT diameters compared with amlodipine therapy. The addition of simvastatin to losartan therapy further decreased the steatosis degree and the SAT and VAT diameters. Losartan and simvastatin combination significantly improved the hepatic steatosis indices compared with amlodipine and simvastatin combination [123]. Glucagon-like peptide-1 (GLP-1) is an incretin secreted by L cells in the small intestine in response to food intake. The main roles of GLP-1 are stimulation of glucose-dependent insulin secretion, inhibition of postprandial glucagon release, delay of gastric emptying and induction of pancreatic β-cell proliferation. Treatment with incretin modulators, GLP-1 analogs and dipeptidyl peptidase-4 inhibitors reduces weight gain, minimizes hypoglycemia, decreases inflammation and is cardioprotective. Several trials evaluating the effects of exenatide (GLP-1 analog) in patients with diabetes revealed that treatment lead to significant improvements of hepatic steatosis and hepatic markers, such as elevated liver enzymes, and other cardiovascular risk factors [124–126]. The addition of pioglitazone to exenatide therapy was associated with a significantly greater reduction in hepatic fat, a greater increase in circulating adiponectin in patients with T2DM as compared to the therapy with pioglitazone alone; moreover, the association therapy patients showed lack of a significant alteration in body weight as compared to the weight gain associated with pioglitazone therapy. The ability of exenatide to reduce appetite and ameliorate thiazolidinedione-induced weight gain may explain the greater reduction in hepatic fat content observed with combined exenatide and pioglitazone therapy [127]. Ursodeoxycholic acid (UDCA) has hepatoprotective and anti-apoptotic effects which could be beneficial in patients with NASH. After ingestion, UDCA becomes the principal bile acid in the liver, replacing more toxic hydrophobic bile acids and stimulating secretion of bile acids. Since steatotic hepatocytes are more susceptible to necrosis and generate increased quantities of hydroperoxides resulting in increased oxidative stress when exposed to hydrophobic bile acids, UDCA may potentially ameliorate this type of liver injury [128]. In addition to these effects, UDCA stabilizes mitochondrial membranes and inhibits caspase activation, having the net effect of inhibiting apoptosis, which has been noted to be increased in patients with NASH. Despite preliminary evidence suggesting a possible favourable effect of UDCA on liver injury in patients with NASH, a large randomized controlled trial found no difference in biochemistry or liver histology with UDCA or placebo after 2 years of treatment [128]. Although high-dose UDCA has anti-apoptotic and antioxidative properties, it does not appear to normalize aminotransaminase levels in patients with NASH [129]. In contrast, another trial showed that treatment with high-dose UDCA was safe and improved aminotransferase levels, serum fibrosis non-invasive markers (FibroTest) and metabolic parameter [130]. New interest is focused on the farnesoid X receptor (FXR), a transcription factor that is fundamental for different pathways regulating lipid metabolism in the liver. FXR is activated by physiologic concentrations of biliary acids and by its agonists that have been suggested as therapeutic tools against NAFLD, insulin resistance and liver fibrosis. Obeticholic acid (OCA, 6α-ethyl-chenodeoxycholic acid) is a semi-synthetic derivative of the primary human bile acid chenodeoxycholic acid, the natural agonist of the farnesoid X receptor that in a 6-week clinical trial was well tolerated, increased insulin sensitivity and reduced markers of liver inflammation and fibrosis in patients with type 2 diabetes mellitus and non-alcoholic fatty liver disease [131]. Orlistat (Xenical) is a natural lipase inhibitor, impedes then dietary triglyceride hydrolysis in the gut and reduces the absorption of dietary fat by 30%, improving IR and lipid profile. In the liver, the suggested role of orlistat is in reducing fatty infiltration and improving hepatic fibrosis, with significant improvement in serum levels of ALT, AST and lipids [132]. But in another trial, orlistat did not enhance weight loss, improve liver enzymes and IR, or histopathology when associated to a 1,400 kcal/day diet plus vitamin E [133]. Furthermore, orlistat, alone or in combination with ezetimibe had more favourable effect on LDL and additionally improved BMI, transaminase levels and insulin resistance [134]. Pentoxifylline (PTX) is a methylxanthine that inhibits a number of pro-inflammatory cytokines including TNF alpha and may have potential hepatoprotective, anti-fibrogenic effects. This was suggested by *in vitro* studies on HSCs [135] and confirmed by a recent randomized placebo controlled trial that showed improved histological features of NASH, and liver fibrosis, in patients treated with PTX [136]. Since the known role of gut microbiota in the pathogenesis of NAFLD, many study focused their attention on the research of new therapies with probiotics. In animal model, a multistrain preparation composed of *Streptococcus thermophilus* and several species of *Lactobacillus* and *Bifidobacteria* significantly reduced serum and liver triglyceride concentrations. which is associated with a reduction of fat mass. This supports the anti-inflammatory and antioxidative activity of VSL#3 on liver, which is responsible in the preventative effect of the early onset of NASH [137]. Furthermore, in a clinical trial, a tablet of 500 million *Lactobacillus bulgaricus* and *Streptococcus thermophilus* improved liver aminotransferase levels in patients with NAFLD [138]. More studies are needed, but this is a new and fascinating approach to liver disease.

Despite the appropriateness of these clinical researches and trials, also challenging the possible favourable collateral effects of drugs used for other diseases, such as arterial hypertension and diabetes, results are still null or disappointing.

The cornerstone of any approach is still based on healthy nutrition and lifestyle enhancement, also by the promotion of healthy diet paradigms [101, 139–143]. Relationship with adipogenic adenovirus infections [144], other organ disease [145–149] and environment [150, 151] is equally important also for research and exploitation of societal intervention [152, 153] and represents a field of applicative research behind the boundaries of nutrients, nutritional profile and lifestyles, trying to involve wider environmental and multi-organ approaches. Overall, the pathways of the “magic bullets” for managing liver disease and, particularly, NAFLD are not so straightforward. Specific food or beverage and their content nutrients could have an effect on or modify the occurrence and course of liver diseases, acute or chronic, and modulate or counteract the effects of natural or synthetic toxic substances. Preliminary evidence supports the use of several nutraceutical agents in the treatment of liver diseases for which conventional therapies are limited. It is known that in chronic liver disease, as in other conditions, malnutrition occurs due often also to inappropriate nutritional behaviour, bias and counseling, and it is correlated with poorer quality of life and greater mortality outcome. Anorexia, nausea and constipation that frequently accompany liver disease and reduce dietary intake can be present without frank or severe liver disease but exerting unfavourable effects on liver by malabsorption and, as written above, microbiome alterations. There is the evidence that some dietary substances, such as silymarin, a flavonoid derived from the seeds of *Silybum marianum* (milk thistle), are useful in liver disease, probably by preventing lipid peroxidation. Its more active derivative drug, silybin, was associated with improvement in liver enzymes, insulin resistance and liver histology without increases in body weight in NAFLD patients [154], but also this pharmacological approach is still a niche application, which, even anecdotally, is of benefit, in our experience, for several patients.

##### Suboptimal health

Despite it is true that “experience without theory is blind, but theory without experience is mere intellectual play” (Immanuel Kant), translational medicine, from models to practice, needs convincing evidence and is confirmed by practitioners’ verification. The current dissemination of lifestyle and nutritional intervention is more market- than evidence-based, and the use of non-conventional approaches requires a great level of attention and care to their scientific proofs, if any [155]: “a lie gets halfway around the world before the truth has a chance to get its pants on” (Winston Churchill). Everybody understands the reasons of research. Also in medicine, research is a risky process: in these subsets, “chance is always powerful and it is necessary that the hook always be cast; in the pool where you least expect it, there will be fish” (Ovid). Nonetheless, “chance favors the prepared mind” (Louis Pasteur), and by this, innovation and discoveries ensue. So many facts are known about fatty liver, and certainly new information and ideas are welcome. But current facts, which address professional behaviours, are more driven by epidemiology than by molecular biology knowledge.

**Figure 8:**
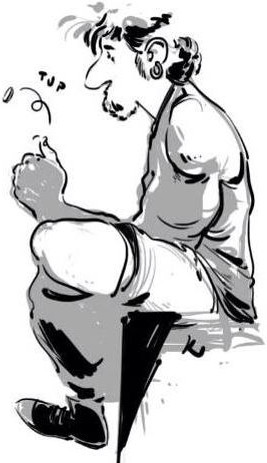
**Is the clinic of NAFLD a hazard?** It is not true that the choices in the management of NAFLD are scarcely relevant, so that can be taken by chance addressing more to one aspect or the other. A great level of attention should be present in the assessment of established or new knowledge: the drift caused by inconsistent articles and reports, and scarcely supported conclusions, prevents the dissemination of more robust studies and valid approaches. A lie gets halfway around the world before the truth has a chance to get its pants on. The clinic of liver disease is a comprehensive approach, and the concept that one special issue can be the definite answer for diagnosis or therapy is not achieved. “Luck is not chance, it’s toil; fortune’s expensive smile is earned”.

A great level of attention should be present in the assessment of established or new knowledge: the drift caused by inconsistent articles and reports, and scarcely supported conclusions, prevents the dissemination of more robust studies and valid approaches. It is not true that the choices in the management of NAFLD are scarcely relevant, so that can be taken by chance addressing more to one aspect or the other (Figure 8). The clinic of liver disease is a comprehensive approach, and the concept that one special issue can be the definite answer for diagnosis or therapy is not achieved. “Luck is not chance, it’s toil; fortune’s expensive smile is earned” (Emily Dickinson). There is the need of a wider awareness of ethics in medical research. It is not only a matter of science integrity and research misconduct, both problems with obvious costs which are difficult to measure [156]; it is also a more general matter of national European research guidelines which are so hard to find, that it is not likely can they then serve as a framework for researchers. Moreover, how can researchers cooperate in international research projects with such diversity in guidelines? European countries are not yet united when it comes to guiding scientific integrity [157], and European rules of funding are providing a great help toward a greater rationalization of this context. The task of assessing futility and—differently—quality and innovation of ideas and approaches is still and even more than previously in the minds of expert evaluators, who are in the most appropriate position to independently evaluate independent research. The visibility of this independence can be provided by several behaviours, including also active interventions against publications questionable for methodology and unsupported conclusion. In this sense, the work in the field of 4Ps and NAFLD is a model of the articulation of basic translational research and evidence-based clinical approaches and outcomes.

There is also the need of a deeper awareness of ethics of competences in health professionals. The articulation of knowledge, expertise and skills of medical doctors, dietitians, health psychologists and sport and physical exercise graduates is the necessary strategy for achieving realistically beneficial lifestyle changes. These interventions can be greatly personalized with specific procedure, such as cardiorespiratory assessment tests, which are currently used mostly in cardiac and respiratory rehabilitation for addressing the work of physiotherapists. An appropriate intervention on NAFLD and obesity, such as equally on hypertension, diabetes, depression and other conditions for which the benefit of appropriate increase of physical activity is recognized, includes the need of an adequate involvement of certified health professionals. Suboptimal health status (SHS) is considered to be an intermediate status between disease and health, and is characterized by a decline in vitality, in physiological function and in the capacity for adaptation [158], and is a more general condition which is well, comprehensively and very diffusely identifiable in the perverse liaison of obesity, fatty liver and unhealthy environment, social conditioning and lifestyle [43, 101, 140–143, 150, 151]. It is recognized that the combination of high prevalence of multiple risk factors and low levels of awareness of their suboptimal health status herald a looming epidemic of life-threatening diseases [159]. Lifestyle is one of the most important factors affecting health status and its perception, particularly in youngsters, and relationship of body size, shape and appearance perception with actual body measurements, such as weight, is strongly influenced by media and, namely, by fashion’s trends, in youngsters [160, 161] and in workers [162]. For these reasons, multidimensional assessment and intervention aimed to favorable modifications of lifestyles including food quality [163], sedentary life [164] and healthy sleep [165], gender issues and gender discrimination, alcohol, illicit drugs, bullying and nightlife habits [166], are likely to move suboptimal health toward better health profiles.

The concept of 4Ps in NAFLD is summarized by the concise sentence of Albert Einstein: “The devil has put a penalty on all things we enjoy in life. Either we suffer in health or we suffer in soul or we get fat”. This is quite hyperbolic, of course, but something is true: the task of medical research and intervention is to make possible to enjoy life also without things that make sufferance in health and souls and which increase body fat. This model is suitable for addressing prevention and useful for monitoring improvement, worsening and adherence with non-invasive imaging tools which allow targeted approaches.

## Conclusions

The road for addressing straight-to-the-point predictive, preventive and personalized medicine in obesity, fatty liver and fibrosis epidemics must take into account the multifactorial pathogenesis of NAFLD. Diagnosis is confirmatory by imaging (ultrasound) and of exclusion again by imaging and the absence of relevant signs of inflammation attributable to liver disease. Numerous treatments for various contributory risk factors have been proposed, but there is no single validated therapy or association recommended for all cases irrespective of the primary NAFLD risk factors. A predictive, preventive, personalized and participatory medicine approach must take into account several perspectives related to this subject; a greater focus to best evidences and established practice is suggested to get sustainable and cost-benefit valuable outcomes by participatory interventions (see list below). The strategic components can be enhanced within comprehensive research projects focused to societal issues and innovation, market appeal and industry development, cultural acceptance and sustainability.

Below are expert recommendations for the management of obesity and fatty liver:

- wider use of expertise in morphologic non-invasive tools and mainly ultrasound for liver and fat cell mass and also with early undergraduate and postgraduate teaching and training;
- use of knowledge and skills, with availability of suitable software, in nutritional and physical activity assessment and prescription according to validated guidelines on lifestyles and also with early undergraduate and postgraduate teaching and training;
- articulated professional staff, patient-friendly, with pro-active frames using health psychology empowerment, adherence and self-efficacy enhancement;
- research development strategies, warranting evidence-based application and reappraisal of the current practice and knowledge;
- collaboration with non-profit patient’s associations, media, various institutions (schools, cities, trade and work organization) with the aim of better managing societal issues and enhancing participatory medicine.

The basis of participatory medicine is a greater widespread awareness of a condition which is both a disease and an easy documented and inclusive clue for associated diseases and unhealthy lifestyle. NAFLD, like it is, is a suitable target for addressing prevention and is useful for monitoring improvement, worsening and adherence by non-invasive imaging tools which allow targeted approaches.

The road to increasing knowledge of epidemiology, mechanisms and biological signatures implies a coherent, articulated and widespread increase of medical skills in non-invasive diagnostic tools, particularly clinical ultrasound, and in intervention strategies effective on lifestyle change. The latter include health psychology and nutritional and physical exercise prescription expertise disseminated by continuous medical education but, more important, by concrete curricula for training undergraduate students. It is possible and recommended to do it by formal teaching of ultrasound imaging procedures and of practical lifestyle intervention strategies. Guidelines and requirements of research project funding calls should be addressed also to NAFLD and allied conditions and should encompass the goal of training by research and the inclusion of participatory medicine topics.

## Authors’ information

GMT is an EPMA Institutional Member (University of Catania) Coordinator of EPMA Italy, Associate Editor for Clinical Nutrition, Health Psychology and e-Learning and ICT in Health Science, the coordinator of the Research and Innovation Project and Design Planning of the University Hospital of Catania and the Director of the Postgraduate School of the Master in e-Learning and ICT in Medicine and Health Sciences. DC is the Director of the Postgraduate School of the Master in Clinical Ultrasound.

## Abbreviations

^1^H-MRS: proton magnetic resonance spectroscopy
ACEIs: angiotensin-converting enzyme inhibitors
ALT: alanine aminotransferase
APRI: AST/platelet ratio index
ARBs: angiotensin II receptor blockers
ARFI: acoustic radiation force impulse
AST: aspartate aminotransferase
BLS: bright liver score
BMI: body mass index
CAP: controlled attenuation parameter
CEUS: contrast-enhanced ultrasound
CK-18: cytokeratin 18
CT: computed tomography
ELF: enhanced liver fibrosis
FFA: free fatty acids
GLP-1: glucagon-like peptide-1
H&E: haematoxylin and eosin
HIF-1α: hypoxia-inducible factor 1 alpha
HSCs: hepatic stellate cells
HTGC: hepatic triglyceride content
IH: intermittent hypoxia
IL-6: interleukin-6
IR: insulin resistance
MFB: myofibroblast-like cells
MRE: magnetic resonance elastography
MRI: magnetic resonance imaging
MS: metabolic syndrome
MUFA: monounsaturated fatty acids
NAFLD: non-alcoholic fatty liver disease
NAS: NAFLD activity score
NASH: non-alcoholic steatohepatitis
NPV: negative predictive value
NT: NashTest
OSA: obstructive sleep apnea
PAI-1: plasminogen activator inhibitor-1
PDGF: platelet-derived growth factor
PNPLA3: patatin-like phospholipase domain-containing 3
PPAR: peroxisome proliferator-activated receptor
PTX: pentoxifylline
RAS: renin-angiotensin system
ROS: reactive oxygen species
SAT: subcutaneous adipose tissue
SIBO: small intestinal bacterial overgrowth
SREBP-1: sterol regulatory element-binding protein-1
ST: SteatoTest
TG: triglycerides
TGF-β1: tumour growth factor beta-1
TNF-α: tumour necrosis factor-alpha
TZDs: glitazones
UDCA: ursodeoxycholic acid
US: ultrasound
VAT: visceral adipose tissue
VF: visceral fat
VLDL: very-low-density lipoprotein
WC: waist circumference
WHR: waist-to-hip ratio
ω-3 PUFAs: omega-3 polyunsaturated fatty acids.
